# Prognosis of colorectal cancer patients is associated with the novel log odds of positive lymph nodes scheme: derivation and external validation

**DOI:** 10.7150/jca.38180

**Published:** 2020-01-16

**Authors:** Qing-Wei Zhang, Chi-Hao Zhang, Yuan-Bo Pan, Alberto Biondi, Valeria Fico, Roberto Persiani, Shan Wu, Yun-Jie Gao, Hui-Min Chen, Ou-Min Shi, Zhi-Zheng Ge, Xiao-Bo Li

**Affiliations:** 1 Division of Gastroenterology and Hepatology, Key Laboratory of Gastroenterology and Hepatology, Ministry of Health, Renji Hospital, School of Medicine, Shanghai Jiao Tong University, Shanghai Institute of Digestive Disease, Shanghai, China; 2 Department of General Surgery, Shanghai Ninth People's Hospital, School of Medicine, Shanghai Jiao Tong University, Baoshan, 201999, Shanghai, China; 3 Department of Neurosurgery, Second Affiliated Hospital, School of Medicine, Zhejiang University, Hangzhou, Zhejiang, China.; 4 Dipartimento Scienze Gastroenterologiche ed Endocrino-Metaboliche, Fondazione Policlinico Universitario A. Gemelli IRCCS -Università Cattolica del Sacro Cuore, Roma, Italy Largo F. Vito, 100168 Rome, Italy.; 5 School of Public Health, Shanghai Jiaotong University School of Medicine, South Chongqing Road No, Shanghai 227, China.

**Keywords:** Colorectal cancer, cause-specific survival, overall survival, log odds of positive lymph nodes, multicenter, SEER.

## Abstract

**Background and aim:** To construct proper and externally validate cut-off points for log odds of positive lymph nodes scheme (LODDS) staging scheme in colorectal cancer (CRC).

**Patients and methods:** The X-tile approach was used to find the cut-off points for the novel LODDS staging scheme in 240,898 patients from the Surveillance, Epidemiology and End Results (SEER) database and externally validated in 1,878 from the international multicenter cohort. Kaplan-Meier plot and multivariate Cox proportional hazard models were performed to investigate the role of the novel LODDS classification.

**Results:** The prognostic cut-off values were determined as -2.18, and -0.23 (*P*< 0.001). Patients had 5-year cancer-specific survival rates of 83.8%, 57.4% and 24.4% with increasing LODDS (*P*< 0.001) in the SEER database. Five-year overall survival rates were 77.2%, 55.0% and 26.7% with increasing LODDS (*P*< 0.001) in the external international multicenter cohort. Multivariate survival analysis identified both the LODDS classification, the patient's age, the T category, the M status, and the tumor grade as independent prognostic factors in both two independent databases. The analyses of the subgroup of patients stratified by tumor location (colon or rectum), number of retrieved lymph node (< 12 or ≥ 12), TNM stage III, lymph node-negative also confirmed the LODDS as independent prognostic factors (*P*< 0.001) in both two independent databases.

**Conclusions:** The novel LODDS classification was an independent prognostic factor for patients with CRCs and should be calculated for additional risk group stratification with pN scheme.

## Introduction

The presence of lymph node metastases (LNM) and the number of lymph node metastasis, also called the number of positive lymph node (pN), are robust risk factors in patients with colorectal cancers (CRCs) 1, which may determine subsequent adjuvant therapies and surveillance strategies 2, 3. Based on the number of involved LNM, CRCs could be classified as pN0, pN1 (1-3 tumor invaded LNM), and pN2 (4 and more tumor invaded LNM) cancers 4.

Adequate lymph node histopathological assessment is of significant impact on accurate pN staging but the minimum of recommendations in the literature ranged significantly 5-7. In order to avoid stage migration effect, adequate evaluation of the lymph node status is of great importance and, to date, the widely accepted minimum number of retrieved lymph nodes is 12 8-10. However, it was surprising that the reported numbers of retrieved lymph nodes (rN) in CRCs varied widely in published literatures, median ranging from 6 to 13 11, 12, though precise recommendations and guidelines are available.

Considering inadequate examination of lymph nodes in nearly half of CRC patients, it is urgent to recommend new measures of lymph node status with combination of rN. To the best of our knowledge, two measures, namely lymph node ratio (LNR) 13, log odds of positive lymph nodes (LODDS) 14 have been proposed. Multiple studies have shown superiority of LNR to pN in accurately predicting patient's survival 13, 15. Although LNR seems to be a superior predictor of survival in Stage III colorectal cancer 7, 15-18, the results remained controversial, particularly in CRC with no LNM and inadequate rN. In node-negative CRCs accounting for more than half of CRCs 19, LNR0, same to pN0 classification, does not provide any more additional prognostic evaluation than pN0. In this situation, patients with no LNM may be at high risk of understaging with lack of adequate of rN and incorrect choice of postoperative adjuvant treatments after surgery may be made.

LODDS has been recently proposed as a new prognostic index in CRCs 14, 20-23, showing powerful ability to classify patients into different groups with homogeneous survival, regardless of lymph node status and count. However, different methods were used in different studies to determine their cut-off LODDS values. Three studies used statistical methods to calculate cut-off values in limited numbers of patients 14, 21, 23. Two studies even used arbitrary classification for investigation 20, 22. Though Song's study 23 used the statistical method to calculate cut-off values in relative large number of CRC patients, its cut-off values were not validated in other populations.

In the present study, we aimed to find optimal categorization of LODDS values using X-tile approach in a large population-based database involving 240,898 CRCs and validate our determined LODDS cut-off values in the international multicenter cohort, providing a more precise lymph node staging scheme for patients with CRCs.

## Method

### Surveillance, Epidemiology and End Results Database

Patients with CRCs from Surveillance, Epidemiology, and End Results (SEER) database were created and collected through query to the latest version of the SEER 18 Regs Research data (1973-2014), released in April 2017 with the SEER*Stat 8.3.5 software. The inclusion criteria for selected patients were as follows: 1) Patients aged 18 years old or more diagnosed between 1988 and 2010; 2) Patients with CRCs diagnosed as the only primary cancers without multiple primary cancers elsewhere; 3) Patients with cancers diagnosed microscopically, in whom surgery for primary cancers and regional lymph node resection had been performed with pathological examination of at least one lymph node; 4) International Classification of Diseases for Oncology third edition (ICD-O-3) codes were used as 8010-8231 and 8255-8576 for CRCs; 5) Patients with active follow-up for at least 2 months. Patients were excluded if they received radiotherapy before surgery or CRC was not the only one primary carcinoma or the number of retrieved lymph nodes and positive lymph nodes were missing.

For SEER database, cancer-specific survival (CSS) was defined as death due to CRC and OS was defined as death regardless of any causes. The primary outcome was CSS with OS and CSS considering competing death due to non-CRC death as the secondly outcome. Survival time was defined as the time from diagnosis to the date of death or last contact or Nov 2016.

Since SEER database is public-use data, no institutional review was required, and we have been allowed to access SEER database for only research using the private SEER ID (zhangqw).

### International multicenter cohort

An independent international multicenter cohort from three medical centers was used as validation group using the same inclusion and exclusion criteria. Patients who underwent colectomy for histopathological confirmed CRC from February 2004 to March 2017 in Renji Hospital of of Shanghai Jiao Tong University, Shanghai Ninth People's Hospital of Shanghai Jiao Tong University and from January 2004 to April 2017 in Catholic University of Rome were selected. The follow-up time in the two Chinese hospitals was every 6 months until death or study ended (30^th^ March 2018), except for those lost to follow-up. For patients in the Catholic University of Rome, the patients were followed up until death or study end (30^th^ April 2018) except for those lost to follow-up according to the European Society of Medical Oncology guidelines 24.

This study was approved by the Institutional Review Board in the all participating hospital.

### Definitions of node staging scheme

Two values, namely pN and the number of negative lymph node (nN), were needed to calculate LODDS values. The pN value indicates the number of positive lymph node. rN value is defined as the number of retrieved lymph nodes for histological examination of lymph node metastasis status. The nN value is the absolute number of negative lymph node, which is calculated by subtracting pN value from the total number of rN value. The LODDS value is defined as log_e_ ([pN + 0.5]/ [nN + 0.5]). As a continuous variable, LODDS values are then classified as a novel five-subgroup LODDS classification using proper cut-off points using the X-tile 25.

### Statistical analysis

For descriptive statistics, the absolute number with proportion for categorical variable, mean and standard deviation for continuous variable with Gaussian distribution and median and interquartile range (IQR) for continuous variable with non-normally distribution were used respectively. The chi-square test for categorical variable, Student's t-test for continuous variable with Gaussian distribution and the nonparametric Kruskal-Wallis rank sum test for continuous variable with non-normally distributed data were used for comparisons among different patient groups respectively. The above descriptive statistics and exploratory comparisons were done using CBCgrps package 26.

For survival analysis, Kaplan-Meier method was performed to calculate and show survival rates in different patient groups with log-rank test used for statistical comparisons. Meanwhile, multivariate Cox regression models with variable selection procedures were used to explore potential risk factors associated with patient's survival. Besides, cumulative probability of CRC-specific death and multivariate regression modeling of subdistribution functions in competing risks were also performed for sensitivity analysis for our findings using cmprsk package 27. Five-year survival rate and hazard ratios (HRs) were calculated with 95% confidence intervals (CIs).

To determined optimal categorization of LODDS values, X-tile technique was used to define the optimal cut-off points by the log-rank test 25. Firstly, X-tile technique would divide the population into low-, medium- and high-level LODDS value by every possible cut-off. Then, survival between all possible divisions according to LODDS values were tested by the log-rank test. Finally, the optimal LODDS cut-off would be selected by selecting the highestχ^2^ value.

Statistical analyses and plotting graphics were conducted using R software package (version R-3.4.3, the R Foundation for statistical computing). All statistical comparisons were considered significant with *P*< 0.05.

## Results

### Clinical characteristics of patients from SEER database and the international multicenter cohort

With defined inclusion and exclusion criteria (Supplementary Figure 1), a total of 240,898 patients with CRCs were finally identified from the SEER database. As shown in the Table 1, the median number (IQR) of retrieved lymph nodes was 12 (7, 18) for the total patient group. The median number (IQR) of continuous LODDS value for the total patient group was -2.51 (-3.3, -1.21) and the follow-up time was 65 (26, 116) months.

With the same inclusion and exclusion criteria as SEER database, we identified 1,878 patients from the international multicenter cohort. In the international multicenter cohort, the median number (IQR) of retrieved lymph nodes was 10 (5, 14) for the total patient group. The median number (IQR) of continuous LODDS value for the total patient group was -2.51 (-3.3, -1.21) and the follow-up time was 48 (21, 75) months.

The remaining clinicalpathological characteristics for SEER database and the international multicenter cohort could be seen in the Table 1.

### Clinical characteristics and survival rate among different novel LODDS group: derivation and validation

The X-tile analysis finally identified optimal thresholds of LODDS. The novel LODDS group classified by the cut-off values of -2.18, -0.23 showed the highestχ2 value for the CSS. Therefore, a novel LODDS classification subgroup was determined in this study using the above LODDS cut-off points: LODDS1 (-2.18 or less), LODDS2 (more than -2.18 to -0.23) and LODDS3 (more than -0.23).

**Figure 1:**
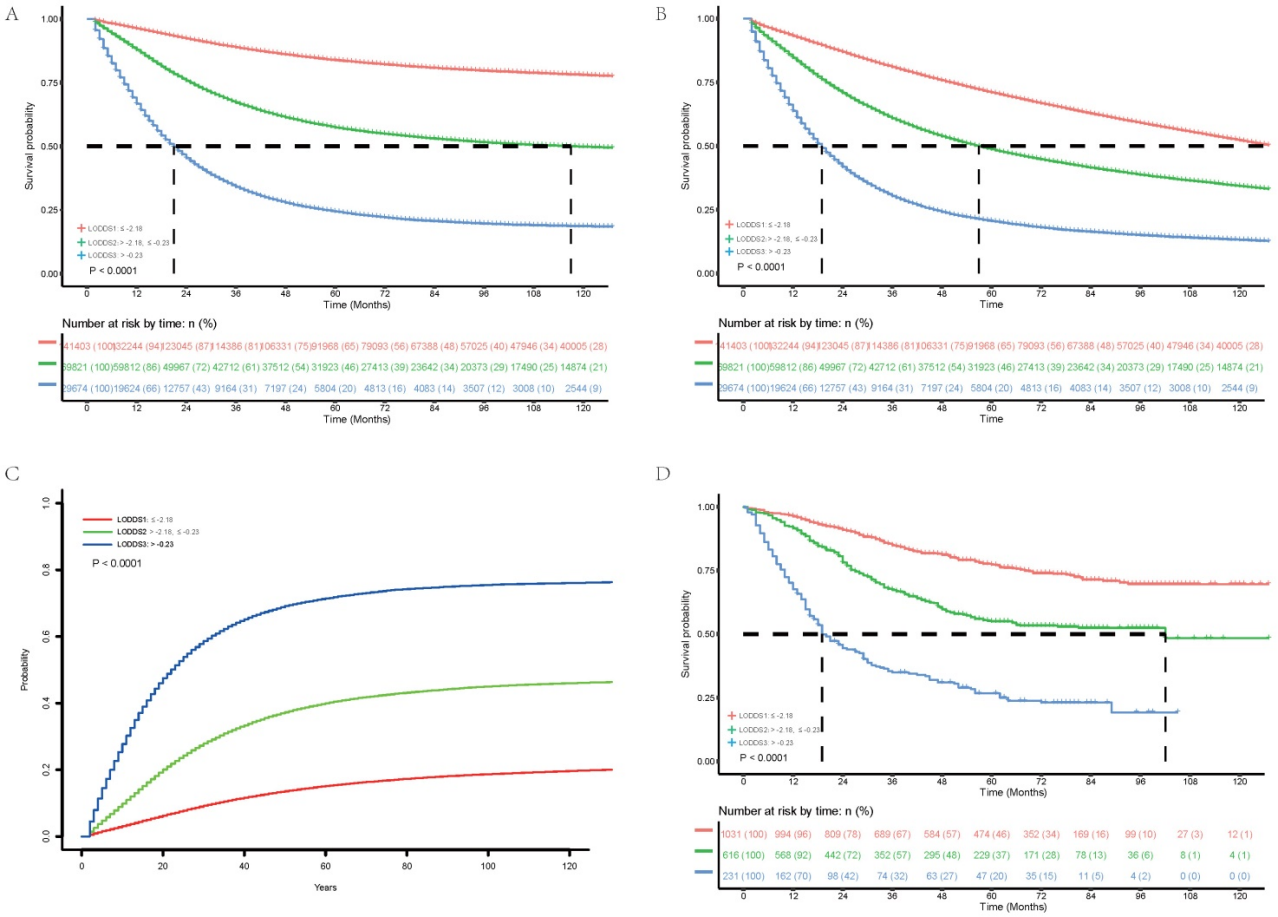
** Cancer-specific survival (A),** overall survival **(B)** and cumulative probability of Cancer-specific death **(C)** of 240, 898 patients in the Surveillance, Epidemiology, and End Results database and overall survival **(D)** of 1, 878 patients in the international multicenter cohort stratified according to the log odds of positive lymph nodes.

We explored association between LODDS classification subgroup and clinical and histopathological characteristics. Results (Table 2) showed positive correlation of LODDS classification with T stage (*P*< 0.001), N stage (*P*< 0.001), M stage (*P*< 0.001), pN (*P*< 0.001) and negative correlation with nN (*P*< 0.001), which supported rationality of the novel LODDS group. Of 240,898 patients analyzed, 141,403 (58.70%) patients were classified as LODDS1 group with 5-year CSS of 83.8% (83.6%-84.0%), 69,821 (28.98%) patients were classified as LODDS2 group with 5-year CSS of 57.4% (57.0%- 57.8%), 29,674 (12.32%) patients were classified as LODDS3 group with 5-year CSS of 24.4% (23.9%-24.9%). Therefore, increased LODDS classification had significant positive association with poor CSS (Figure 1A, *P*< 0.001). Besides, increased LODDS classification had significant positive association with poor OS (Figure 1B, *P*< 0.001) and higher cumulative probability of cancer-specific death (Figure 1C, *P*< 0.001).

We next validated the novel LODDS classification in the international multicenter cohort. As is shown in the Table 2, LODDS classification also showed positive correlation with T stage (*P*< 0.001), N stage (*P*< 0.001), M stage (*P*< 0.001), pN (*P*< 0.001) and negative correlation with nN (*P*< 0.001) in the international multicenter cohort, which was consistent with findings in SEER database. Of 1,878 patients analyzed, 1,031 (54.90%) patients were classified as LODDS1 group with 5-year OS of 77.2% (74.4%-80.1%), 616 (32.80%) patients were classified as LODDS2 group with 5-year OS of 59.2% (54.3%-64.5%), 231 (12.30%) patients were classified as LODDS3 group with 5-year OS of 45.9% (39.0%-54.2%). Therefore, increased LODDS classification had significant positive association with poor OS (Figure 1D,* P*< 0.001).

We further explored whether the established LODDS classification could classify patients into groups with homogeneous survival. As is shown in the Table 3, there were no patients with N0 disease had an LODDS larger than -0.23. However, within the N0 subgroups, one can see a difference in 5-year OS between patients with LODDS1 (72.0% in the SEER database and 77.1% in the international multicenter cohort) and patients with LODDS2 (64.6% in the SEER database and 62.9% in the international multicenter cohort). These difference were highlighted in Table 3 and show the importance of the stratification of patients by our novel established LODDS classification.

### Multivariate Cox analysis: role of novel LODDS classification in patients' survival

**Figure 2:**
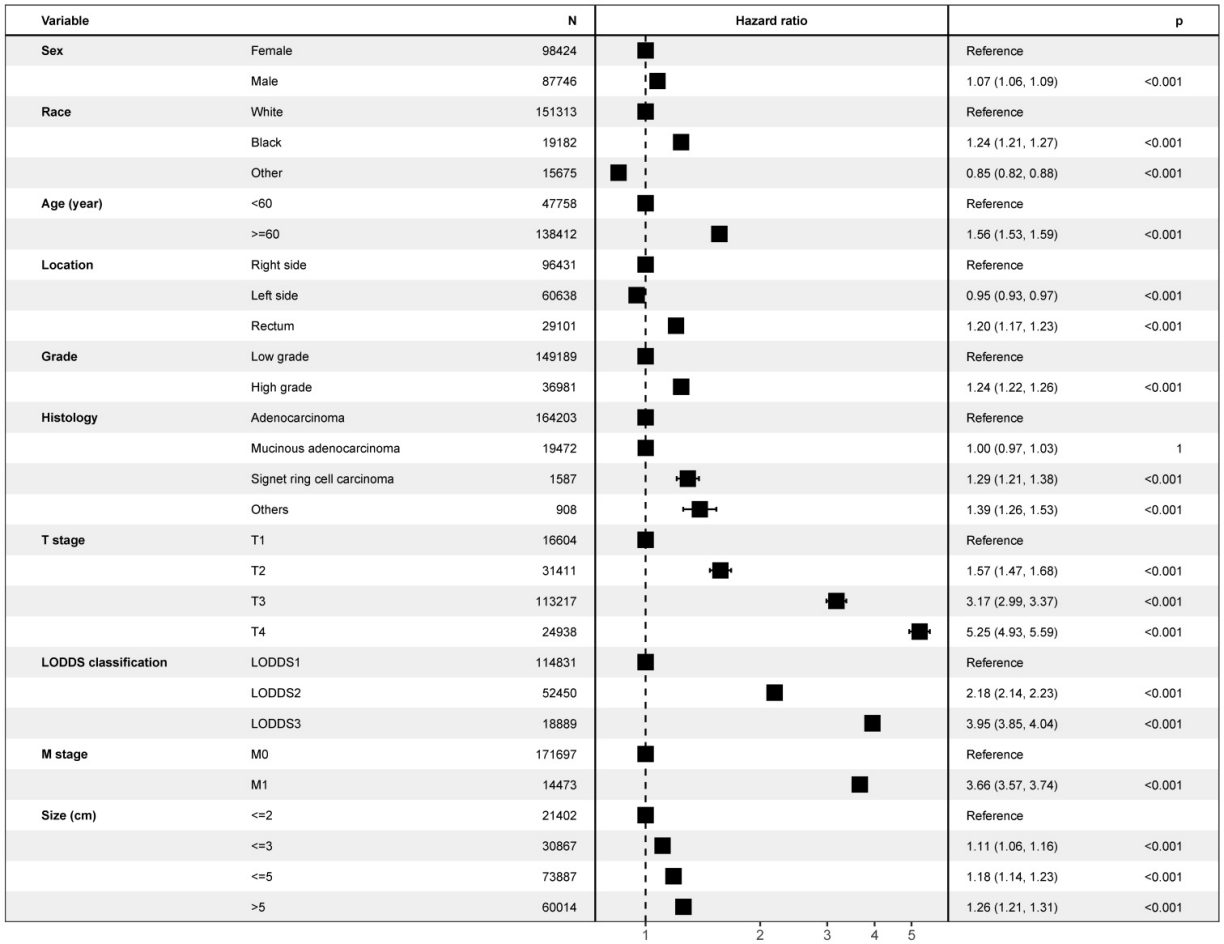
Forest plot showing results of multivariate Cox regression model for exploring potential risk factors with inclusion of covariate log odds of positive lymph nodes for cancer-specific survival in 240, 898 patients of the Surveillance, Epidemiology, and End Results database.

**Figure 3:**
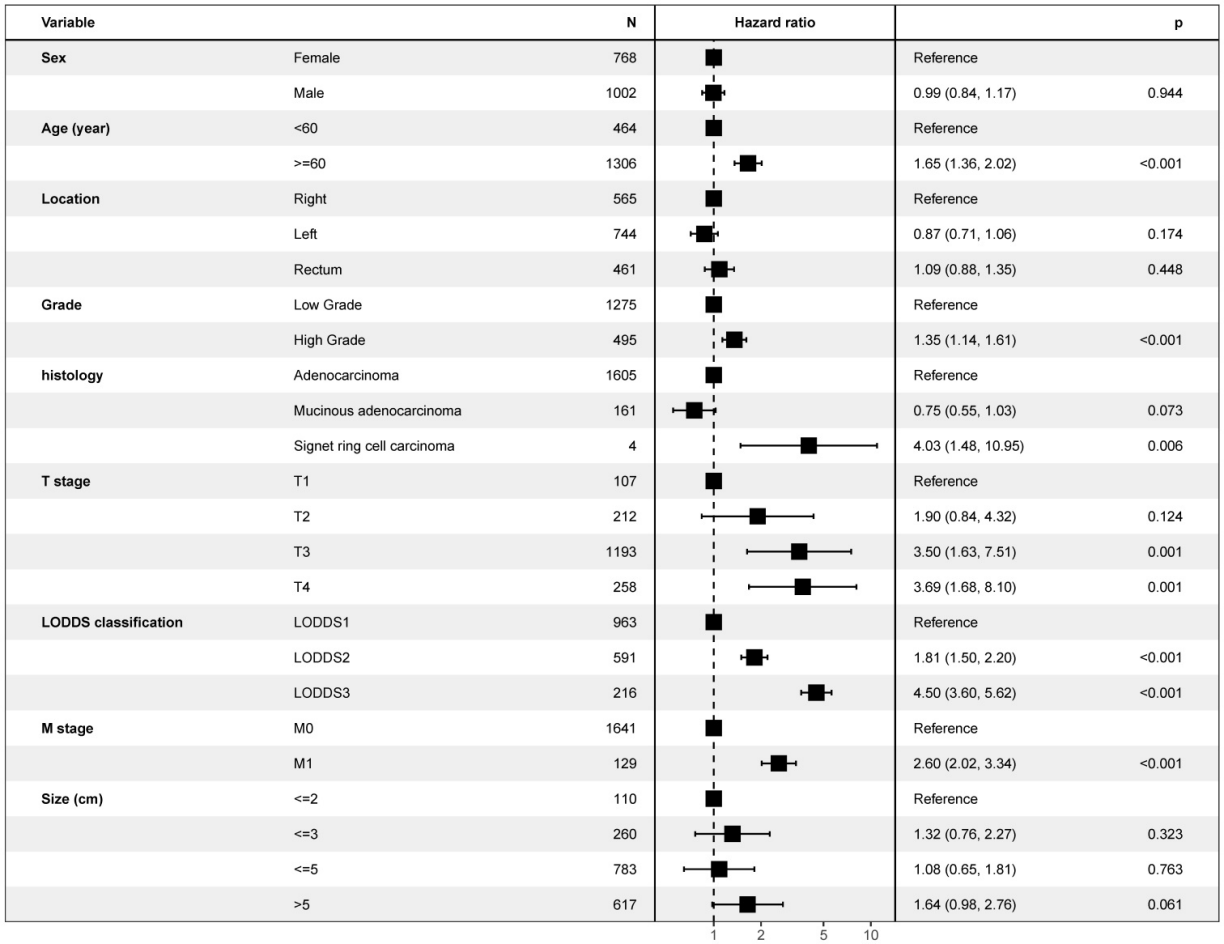
Forest plot showing results of multivariate Cox regression model for exploring potential risk factors for overall survival in 1,878 patients of the international multicenter cohort.

As is shown in the Figure 2, multivariate Cox model, which included LODDS classification and all potential risk factors, identified the LODDS classification (*P*< 0.001), sex (*P*< 0.001), race (*P*< 0.001), age (*P*< 0.001), tumor location (*P*< 0.001), grade (*P*< 0.001), histology (*P*< 0.001), T stage (*P*< 0.001), M stage (*P*< 0.001), and tumor size (*P*< 0.001) as independent prognostic factors. Similar results could be obtained using Multivariate Cox analysis with OS as outcome (Supplementary Figure 2) and multivariate regression modeling of subdistribution functions in competing risks (Supplementary Table 1). As is shown in the Figure 3, it was validated in the international multicenter cohort that LODDS classification, and all prognostic factors were identified as independent prognostic factors.

**Figure 4:**
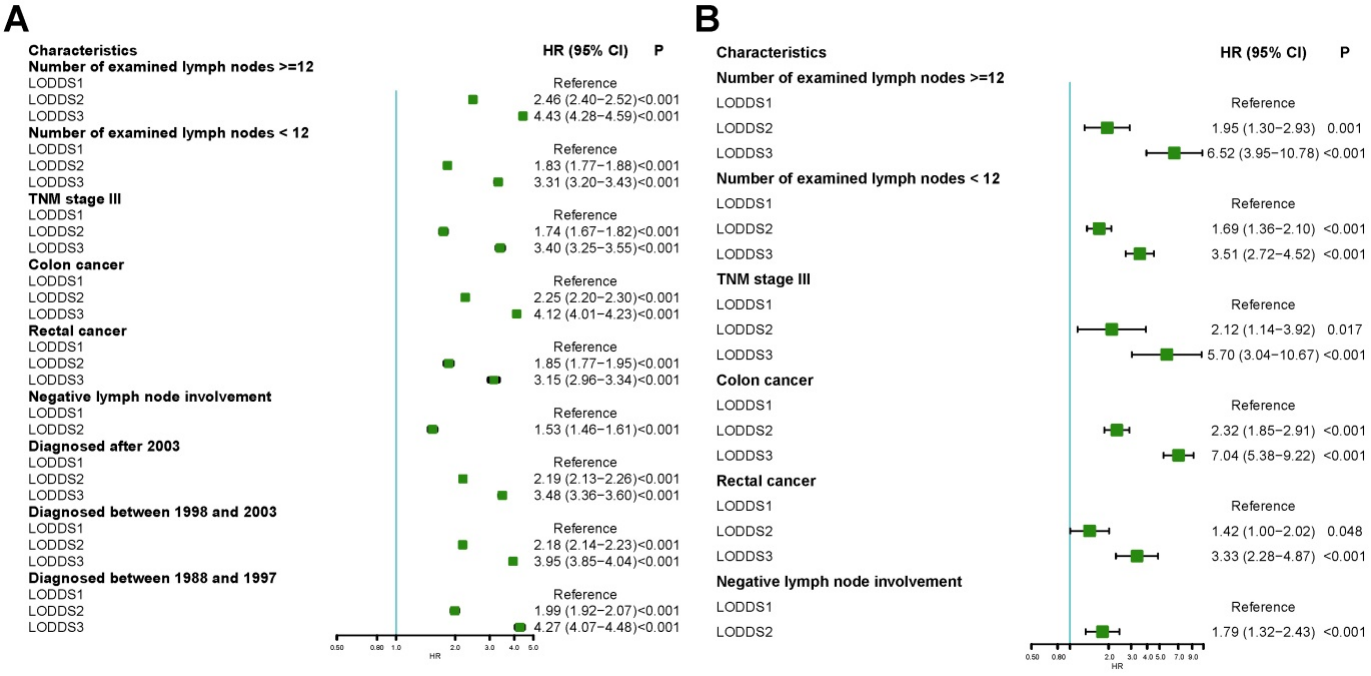
Forest plot showing subgroup analyses results of multivariate Cox regression model stratified by different characteristics in the Surveillance, Epidemiology, and End Results database and international multicenter database.

We next analyzed whether LODDS classification was also an independent risk factors in different subgroups using multivariate Cox regression in the SEER database and the international multicenter database. As is shown in Figure 4A, LODDS classification was identified as an independent risk factor in CRC with number of examined lymph nodes >=12, number of examined lymph nodes <12, 7^th^ TNM stage III, colon cancer, rectal cancer or cancer without lymph node involvement. Similar results could be obtained in the international multicenter cohort (Figure 4B).

Studies have shown that pN category did not show powerful prognostic impact in patients with TNM stage III colorectal cancers 15, 28.

## Discussion

In this study, we presented a population-based analysis of 240,898 patients in SEER database and international multicenter analysis of 1,878 patients in three medical centers. Our analyses showed that LODDS was a powerful prognostic factor for CSS or OS in patients with CRCs in both SEER database and international multicenter cohort. We identified cut-off values -2.18 and -0.23 for LODDS classification. We demonstrated that the novel LODDS classification had significant prognostic impact on CSS or OS in SEER database and the present study was the first study to validate prognostic impact of the novel LODDS classification on survival in an independent cohort from 3 medical centers.

Although pN classification of AJCC TNM classification is the most commonly used staging system for CRCs, it only relies on the number of positive lymph node without the number of retrieved lymph node or the number of negative lymph node, which are also associated with survival 7, 13, 29. Therefore, only when the rN is 12 or more, pN category could be regarded as accurate staging 30, 31. However, case with less than 12 rN are not unusual in clinical practice, which lead to development of new lymph node staging schemes incorporating all the two lymph node information in one single variable. Among the schemes, LNR and LODDS are most promising classifications 13, 14. During the last one decade, multiple studies have poured out that LNR was comparable and even superior to that of well-established prognostic factors, such as TNM classification, in CRCs 13, 15. Unfortunately, LNR has some drawbacks: it do not provide any meaningful information in node-negative CRCs; it do not predict survival well in patients without adequate rN 13; it also cannot discriminate survival difference among patients with all lymph nodes invaded. The other scheme LODDS could solve the above mentioned drawbacks of LNR.

To date, several groups have reported the prognostic impact of LODDS in colorectal cancer (Supplementary Table 2) 14, 20-23. Of them, 3 studies used statistical methods to calculate cut-off values of LODDS for optimal discrimination. The first study 23 used running log-rank statistics to calculate using OS as primary outcome for CRCs in Chinese single-center cohort of limited number of 1297 patients. Our previous study 14 used log-rank test for optimization also using OS in only colon cancers in single-center cohort of limited number of 258 patients. The last study used regression trees technique for classification also using OS in CRCs in Chinese single-center cohort of limited number of 192 patients 21. The other 2 studies 20, 22 even used arbitrary cut-off values for optimization. None of LODDS cut-off values in these studies were validated in another independent cohort. Therefore, there is still lack of proper and accurate LODDS classification. This study was the first study to use the largest number of CRCs for optimization of LODDS using minimal P approach on X-tile software 25 with additionally external validation of determined LODDS cut-off values in an independent international cohort. Different from the above mentioned studies, our study used CSS as primary outcome instead of OS, since prognostic risk factors for CSS could more truly reflect death due to CRCs. Besides, we also tested our cut-off values for OS and CSS under competing risk model with positive results.

One of reason why LODDS is superior to pN or LNR is its prognostic classification of node-negative CRCs patients (pN0 or LNR0). In our study, we found our determined LODDS cut-off values could classify patients with node-negative CRCs into two homogeneous groups with significant prognostic difference, which were consistent with results in other studies 14, 22. In the future, we will evaluate the three lymph node schemes in predicting survival in patients with CRCs to explore whether LODDS is superior to pN or LNR and potential mechanism deeply. Besides, we will try to build a new TNM stage system using our determined LODDS classification for survival optimization of CRCs patients.

Limitation should be discussed in this study. Firstly, it was a retrospective exploratory study based on SEER database and an independent multicenter cohort, clinical and histological characteristics may differ among different registers or hospitals. However, it was actually representative of clinical practice in the real world. Secondly, the follow-up time and number of patients in the independent multicenter cohort were shorter than those in SEER database. It would be better to externally validate our findings in another large multicenter-based cohort or population-based register with longer follow-up time. Thirdly, the study includes patients between 1988 and 2010 when the standard of care for these patients has improved and thus the prognosis has changed. However, we did a subgroup analysis stratified by the time of diagnosis, and results showed patients with LODDS2 or LODDS3 still had poorer prognosis than patients with LODDS1, which indicated that time of diagnosis had limited impact on LODDS classification for prognosis of CRCs. However, our study was different from other studies about LODDS classification in CRCs of which only three 14, 21, 23 used the statistical method to find the optimal cut-off values for LODDS and none tested whether their LODDS classification still had significant impact on prognosis in the other independent validation datasets. We used the largest number of CRCs for optimization of LODDS using minimal P approach on X-tile software. We also used the international multicenter cohort to test whether our established LODDS classification still had significant impact on prognosis in the other independent validation datasets with positive results. In the future, we would also develop a novel prediction model based on our established LODDS classification.

In summary, the present study involved the largest number of CRCs to identify the optimal thresholds of LODDS and was the first study to evaluate the determined cut-off values of LODDS in an independent multicenter cohort. The LODDS classification, with cut-off values -2.18, and -0.23, was an independent prognostic factor in patients with CRCs regardless of tumor location, number of retrieved lymph node, stage III and node-negative cancers. LODDS could improve the prognostic power of current staging systems and should be documented additionally for cancer staging of CRCs.

## Supplementary Material

Supplementary figures and tables.

## Figures and Tables

**Table 1:** Characteristics of patients with colorectal cancer in Surveillance, Epidemiology, and End Results database (N= 240898) and the international multicenter cohort (N= 1878).

		No. of patients (Percent)	
		SEER database	multicenter cohort
Race	White	195333 (81.30%)	NA
	Black	25280 (10.52%)	NA
	Others	19654 (8.18%)	NA
Sex	Female	125827 (52.23%)	811 (43.23%)
	Male	115071 (47.77%)	1065 (56.77%)
Age (year)	Median (IQR)	69 (59, 78)	68 (59, 76)
	< 60	64750 (27%)	489 (26.26%)
	≥ 60	176148 (73%)	1373 (73.74%)
Tumor size (cm)	Median (IQR)	4.40 (3.00, 6.00)	4.50 (3.50, 6.00)
	≤ 2	24020 (11.18%)	116 (6.24%)
	≤ 3	34309 (15.96%)	269 (14.47%)
	≤ 5	85233 (39.65%)	823 (44.27%)
	> 5	71390 (33.21%)	651 (35.02%)
Tumor site	Proximal colon	119078 (50.13%)	604 (32.46%)
	Distal colon	79834 (33.61%)	779 (41.86%)
	Rectum	38642 (16.27%)	478 (25.69%)
Histology	Adenocarcinoma	211146 (87.65%)	1688 (89.83%)
	Mucinous adenocarcinoma	25809 (10.71%)	186 (9.91%)
	Signet ring cell carcinoma	2371 (0.65%)	4 (0.21%)
	Others	1572 (0.98%)	NA
Grade	Well/Moderately differentiated	182915(79.67%)	1327 (71.89%)
	Poorly or undifferentiated	46663 (20.33%)	519 (28.11%)
7^th^ T stage	T1	31113 (14.21%)	113 (6.09%)
	T2	35099 (16.03%)	219 (11.81%)
	T3	124422 (56.82%)	1246 (67.17%)
	T4	28372 (12.94%)	277 (14.93%)
7^th^ M stage	M0	203532 (84.83%)	1739 (92.60%)
	M1	36400 (15.17%)	137 (7.29%)
Total no. of nodes retrieved	Median (IQR)	12 (7, 18)	10 (5, 14)
No. of negative nodes (nN)	Median (IQR)	10 (6, 16)	8 (4, 13)
No. of positive nodes (pN)	Median (IQR)	0 (0, 2)	0 (0, 2)
7^th^ N stage (7^th^ pN)	N0	141131 (58.59%)	1131 (60.22%)
	N1	58625 (24.33%)	496 (26.41%)
	N2	41142 (17.07%)	251 (13.37%)
LODDS	Median (IQR)	-2.51 (-3.3, -1.21)	-2.20 (-3.13, -1.10)
LODDS classification	≤ -2.18	141403(58.70%)	1031 (54.90%)
	-2.18 - -0.23	69821 (28.98%)	616 (32.80%)
	> -0.23	29674 (12.32%)	231 (12.30%)
Follow-up (months)	Median (IQR)	65 (26, 116)	48 (21, 75)

LODDS: log odds of positive lymph nodes; NA: not available; IQR: interquartile range.

**Table 2:** Comparison of log odds of positive lymph nodes distribution with histopathological parameters in the SEER database and international database.

	SEER database										International multicenter database								
	Total		≤ -2.18		-2.18 - -0.23		> -0.23		P		Total		≤ -2.18		-2.18 - -0.23		> -0.23		P
	N (%)		N (%)		N (%)		N (%)				N (%)		N (%)		N (%)		N (%)		
**T stage**									< 0.001										< 0.001
T1	31113(14.21%)		24120(17.72%)		6602(10.75%)		391(1.82%)				113(6.09%)		94(9.19%)		19(3.13%)		0(0)		
T2	35099(16.03%)		27440(20.16%)		6720(10.94%)		939(4.37%)				219(11.81%)		164(16.03%)		48(7.91%)		7(3.11%)		
T3	124422(56.82%)		71960(52.88%)		38341(62.43%)		14121(65.76%)				1246(67.17%)		651(63.64%)		425(70.02%)		170(75.56%)		
T4	28346(12.94%)		12568(9.24%)		9756(15.88%)		6022(28.04%)				277(14.93%)		114(11.14%)		115(18.95%)		48(21.33%)		
**N stage**									< 0.001										< 0.001
N0	141131(58.59%)		126873(89.72%)		14258(20.42%)		0(0)				1131(60.22%)		974(94.47%)		157(25.49%)		0(0)		
N1	58625(24.34%)		14357(10.15%)		38442(55.06%)		5826(19.63%)				496(26.41%)		57(5.53%)		369(59.90%)		70(30.30%)		
N2	41142(17.08%)		173(0.12%)		17121(24.52%)		23848(80.37%)				251(13.37%)		0(0)		90(14.61%)		161(69.70%)		
**rN**	12(7,18)		14(9,20)		11(5,16)		10(6,15)		< 0.001		10(5,14)		11(7,15)		8(3,13)		7(4,11)		< 0.001
**pN**	0(0,2)		0(0,0)		2(1,3)		7(4,10)		< 0.001		0(0,2)		0(0,0)		1(0,3)		4(3,7)		< 0.001
**nN**	10(6,16)		14(9,20)		8(4,13)		3(1,5)		< 0.001		8(4,13)		11(7,14)		6(3,11)		2(1,4)		< 0.001
**M stage**									< 0.001										< 0.001
M0	203532(84.83%)		132809(94.26%)		54777(78.81%)		15946(53.99%)				1739(92.70%)		1002(97.38%)		546(88.64%)		191(82.68%)		
M1	36400(15.17%)		8086(5.74%)		14727(21.19%)		13587(46.01%)				137(7.30%)		27(2.62%)		70(11.36%)		40(17.32%)		

SEER: Surveillance, Epidemiology, and End Results; rN: number of retrieved lymph nodes; pN: number of positive lymph node; nN: number of negative lymph node.

**Table 3:** Five-Year Survival Rates by LODDS

	SEER database					International multicenter database			
	Total	≤ -2.18	-1.06 - -0.23	> -0.23		Total	≤ -2.18	-1.06 - -0.23	> -0.23
**All patients**									
CSS	68.9	83.8	57.4	24.4		NA	NA	NA	NA
OS	58.3	71.0	48.6	20.5		63.2	77.2	55.0	26.7
**N0 patients**									
CSS	84.7	85.3	79.6	NA		NA	NA	NA	NA
OS	71.2	72.0	64.6	NA		74.8	77.1	62.9	NA
**N1 patients**									
CSS	57.2	70.9	55.6	33.7		NA	NA	NA	NA
OS	49.0	62.4	47.3	27.3		51.5	78.2	51.6	32.9
**N2 patients**									
CSS	31.2	60.7	43.4	22.1		NA	NA	NA	NA
OS	27.1	54.2	38.3	18.9		32.6	NA	52.7	23.4
