# Supplementary material for: Prognosis of colorectal cancer patients is associated with the novel log odds of positive lymph nodes scheme: derivation and external validation
Source: J Cancer. 2020 Jan 16;11(7):1702–11. doi: 10.7150/jca.38180 (PMC7052858; doi:10.7150/jca.38180)
Supplement: Supplementary file 1 — Supplementary figures and tables. [file jcav11p1702s1.pdf]

Appendix 1

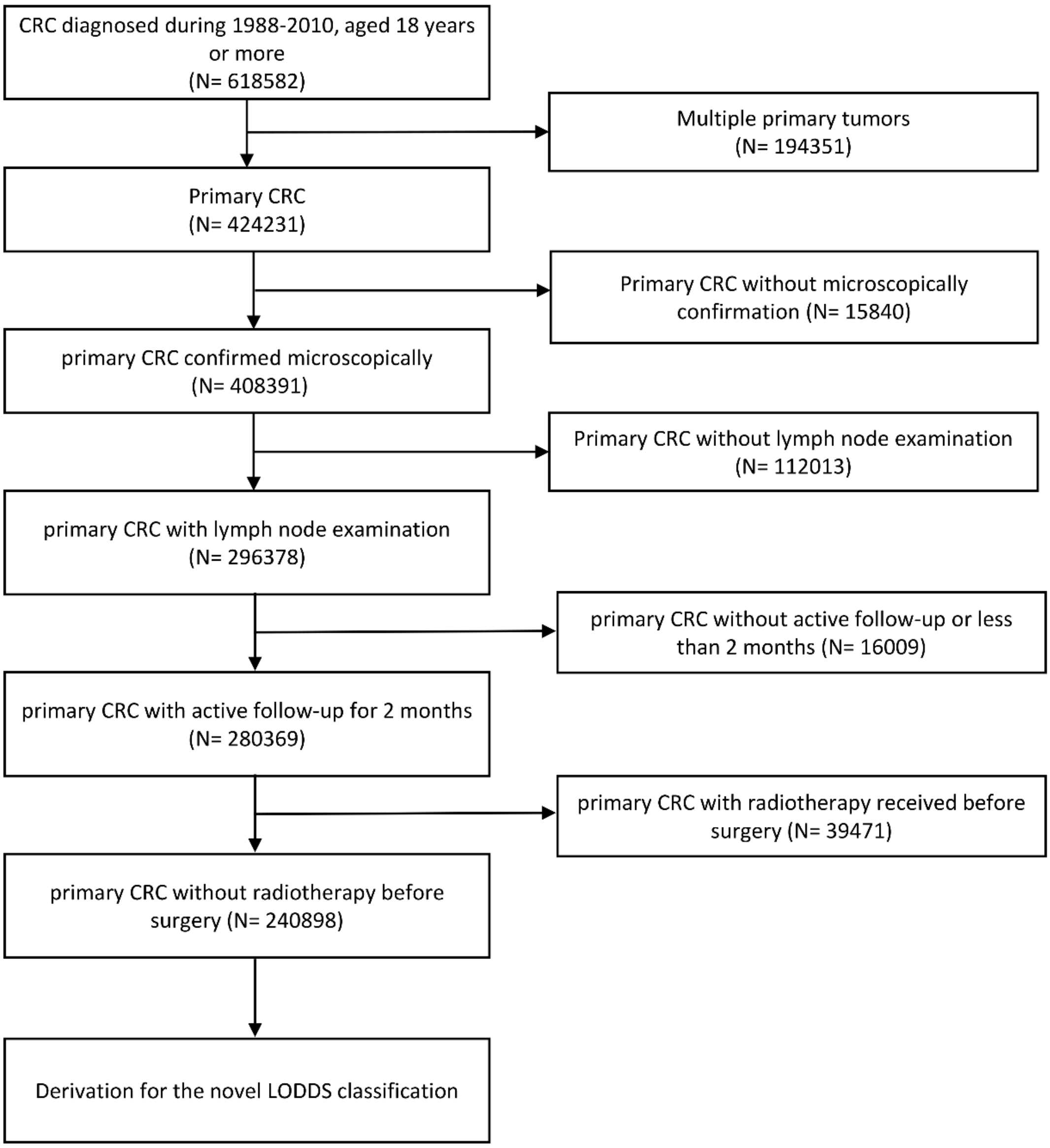

Supplementary Figure 1. Flow chart of selection of patients with colorectal cancer for derivation of the novel log odds of positive lymph nodes classification using the Surveillance, Epidemiology, and End Results database.

Appendix 2

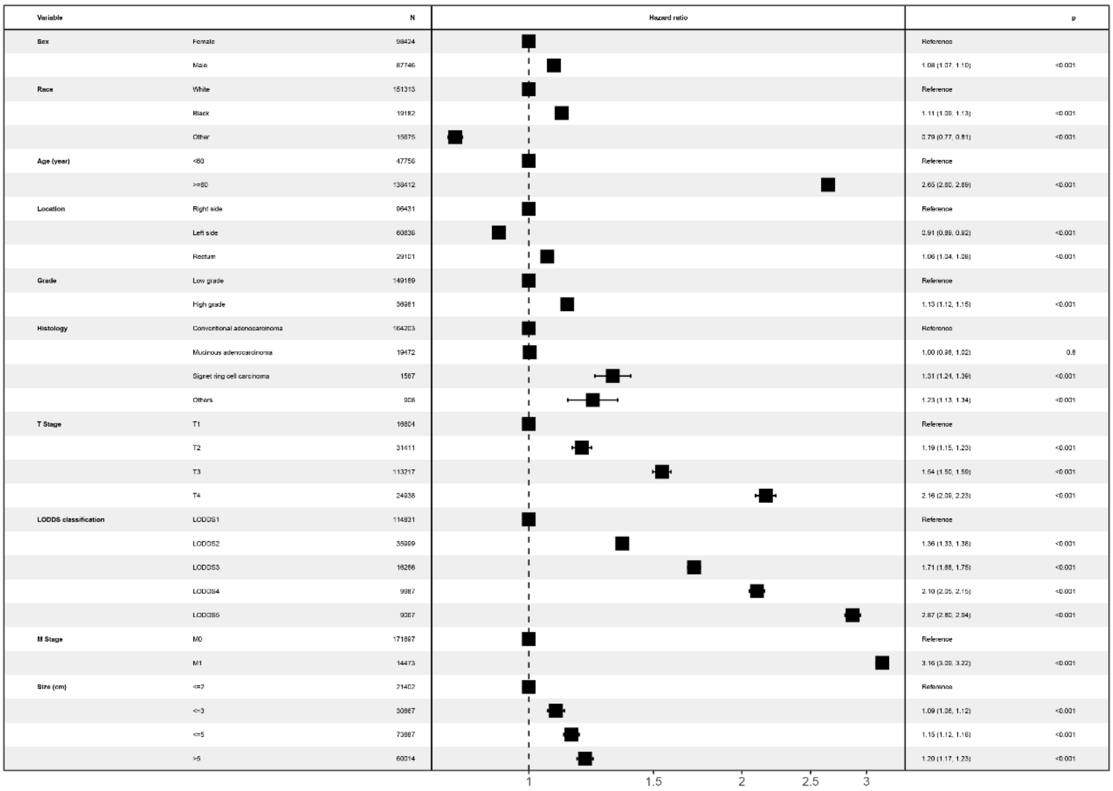

Supplementary Figure 2. Forest plot showing results of multivariate Cox regression model for exploring potential risk factors with inclusion of covariate log odds of positive lymph nodes for overall survival in 240,898 patients of the Surveillance, Epidemiology, and End Results database.

Appendix 3

Supplementary Table 1. Results of competing risks regression with inclusion of the covriate log odds of positive lymph nodes in 240, 898 patients of the Surveillance, Epidemiology, and End Results database.

Factor	SHR	P
**LODDS classification**		
LODDS1	1 (Reference)	
LODDS2	2.14 (2.10-2.19)	<0.001
LODDS3	3.65 (3.56-3.75)	<0.001
**Sex**		
Female	1 (Reference)	
Male	1.06 (1.04-1.07)	<0.001
**Race**		
White	1 (Reference)	
Black	1.21 (1.18-1.24)	<0.001
Others	0.87 (0.84-0.90)	<0.001
**Age**		
< 60	1 (Reference)	
>= 60	1.34 (1.31-1.37)	<0.001
**Location**		
Proximal	1 (Reference)	
Distal	0.97 (0.95-0.99)	0.002
Rectum	1.19 (1.16-1.22)	<0.001
**Grade**		
Low grade	1 (Reference)	
High grade	1.22 (1.19-1.25)	<0.001
**Histology**		
Adenocarcinoma	1 (Reference)	
Mucinous adenocarcinoma	0.99 (0.97-1.03)	0.692
Signet ring cell carcinoma	1.19 (1.10-1.30)	<0.001
Others	1.33 (1.17-1.52)	<0.001
**T stage**		
T1	1 (Reference)	
T2	1.56 (1.46-1.66)	<0.001
T3	3.09 (2.91-3.29)	<0.001
T4	4.97 (4.67-5.30)	<0.001
**M stage**		
M0	1 (Reference)	
M1	3.41 (3.43-3.50)	<0.001
**Size**		
<=2	1 (Reference)	
<=3	1.10 (1.05-1.14)	<0.001
<=5	1.17 (1.12-1.21)	<0.001
>5	1.22 (1.18-1.28)	<0.001

LODDS: log odds of positive lymph nodes; pN: number of positive lymph node.

Appendix 4

Supplementary Table 2. Review of literature including all studies which showed statistical significance of LODDS in colorectal cancer.

Author	Year	No. of patients	Tumor location	Stage	LODDS classification	Statistics
Wang (SEER) (20)	2008	24477	Colon	III	-2.2, -1.1, 0, 1.1	No
Song (23)	2011	1297	Colorectal	I-III	-2.51, -1.68, -0.51, 0.73	Yes
Persiani (14)	2012	258	colon	I-IV	-1.36, -0.53	Yes
Arslan (22)	2014	558	Colon	I-III	-1.36, -0.53	No
Fang (21)	2017	192	Colorectal	I-III	-0.82, -0.57	Yes

LODDS: log odds of positive lymph nodes; SEER: Surveillance, Epidemiology, and End Results database.
